# Green exercise, nature connectedness, and academic burnout: a psychological study based on Chinese university students

**DOI:** 10.3389/fpsyg.2025.1735287

**Published:** 2026-01-08

**Authors:** Tong Liu, Bo Peng, Weisong Chen, Yawei Ren, Hang Hu

**Affiliations:** School of Sports Training, Chengdu Sport University, Chengdu, Sichuan, China

**Keywords:** academic burnout, green exercise, mediation, nature connectedness, structural equation modeling, university students

## Abstract

**Purpose:**

This study investigated the associations between green exercise, nature connectedness, and academic burnout among university students, with a focus on whether nature connectedness mediates the relationship between green exercise and burnout.

**Methods:**

A cross-sectional survey was conducted with Chinese university students. Green exercise, nature connectedness, and three dimensions of academic burnout—emotional exhaustion (EE), cynicism (CY), and reduced personal accomplishment (RPA)—were assessed using validated self-report measures. Data were analyzed with confirmatory factor analysis (CFA), correlation analysis, and structural equation modeling (SEM). Mediation effects were tested using bias-corrected bootstrapping with 5,000 resamples.

**Results:**

Green exercise was positively correlated with nature connectedness (*r* = 0.320, *p* < 0.001) and negatively correlated with EE (*r* = −0.284, *p* < 0.001), CY (*r* = −0.256, *p* < 0.001), and RPA (*r* = −0.299, *p* < 0.001). SEM revealed that green exercise had significant direct effects on EE (*β* = −0.217, *p* < 0.001), CY (*β* = −0.183, *p* < 0.001), and RPA (*β* = −0.212, *p* < 0.001). Indirect effects through nature connectedness were also significant: EE (*β* = −0.126, 95% CI [−0.129, −0.074]), CY (*β* = −0.126, 95% CI [−0.147, −0.086]), and RPA (*β* = −0.131, 95% CI [−0.150, −0.088]). Mediation ratios indicated that 36.7% of the effect on EE, 40.8% of the effect on CY, and 38.2% of the effect on RPA were transmitted via nature connectedness.

**Conclusion:**

The findings indicate that green exercise is a robust protective factor against academic burnout, operating both directly and indirectly through enhanced nature connectedness. Framed within Conservation of Resources theory and stress coping perspectives, these results suggest that outdoor physical activity and a strong sense of connection to nature function as interrelated resources that reduce strain, support resilience, and promote student well-being in higher education contexts.

## 1 Introduction

Academic burnout has become a major mental health concern among university students worldwide, especially in highly demanding educational contexts such as China. Academic burnout is typically defined as a stress-related syndrome comprising three components: emotional exhaustion, cynicism, and reduced personal accomplishment (Maslach and Jackson, 1981; Maslach and Leiter, 2016). Emotional exhaustion refers to extreme emotional and physical fatigue resulting from prolonged inability to cope with academic demands (Khammissa et al., 2022). Cynicism (or depersonalization in academic settings) manifests as detached or negative attitudes toward oneself and others (Ruple et al., 2024). Reduced personal accomplishment reflects decreased efficiency and a loss of sense of achievement in academic work (Cairns et al., 2024). Students experiencing academic burnout often feel exhausted, lose interest in their courses, and question their ability to succeed academically. This burnout not only harms students’ emotional well-being but is also associated with lower academic performance, increased absenteeism, and a higher risk of dropping out (Özhan and Yüksel, 2022; Calcatin et al., 2022). Recent surveys indicate that academic burnout is widespread among Chinese university students, particularly due to intense competition and heavy academic workloads—in some samples, more than half of the students reported moderate to high levels of burnout (Liu et al., 2023; Liu et al., 2024; Kong et al., 2025). These concerning trends highlight the need to identify factors that can alleviate academic burnout and promote students’ resilience. However, existing research has largely focused on individual traits or learning behaviors (Cuevas-Caravaca et al., 2024; Ni et al., 2025), and has paid relatively little attention to the role of environmental and lifestyle factors in the development and mitigation of academic burnout. This leaves an important gap at both theoretical and practical levels.

In burnout theory, chronic academic stressors (such as excessive workload and grade pressure) gradually deplete students’ psychological resources, leading to exhaustion and disengagement (Olson et al., 2025). In contrast, experiences that help students restore resources or cope with stress may contribute to reducing burnout. According to Conservation of Resources theory (COR), individuals strive to preserve and replenish resources to avoid stress, and interventions that provide restoration or support may reduce burnout (Prapanjaroensin et al., 2017; Hobfoll, 2011). Building on this, environmental psychology proposes that the natural environment itself can be regarded as an important source of external resources, and a promising direction lies in the potential therapeutic effects of nature based on environmental psychology (Kaplan, 1995). Exposure to natural environments has been shown to reduce stress and improve mood—this phenomenon can be explained by Stress Reduction Theory (SRT), which posits that natural scenes activate a calming parasympathetic response, and by Attention Restoration Theory (ART), which proposes that gentle contact with nature helps restore depleted cognitive attention (Pasanen et al., 2018; Jimenez et al., 2021). Although COR, SRT, and ART have been widely used to explain general stress, emotional recovery, and cognitive restoration (Liu et al., 2024; Hobfoll, 1989), they have seldom been systematically integrated into the framework of academic burnout, and they are rarely used to explain how university students in high-pressure academic contexts may reduce burnout through nature-related behaviors and nature-related psychological resources. Therefore, in this context, two related constructs are worth examining as protective factors for student well-being: green exercise and nature connectedness.

Green exercise refers to physical activity performed in natural or “green” environments, such as parks, forests, or other outdoor green spaces (Pretty et al., 2005). Unlike exercising in gyms or urban environments, green exercise combines the well-established benefits of physical activity with the psychological benefits of exposure to nature. An increasing body of research suggests that exercising in natural environments provides greater mental health benefits than exercising indoors or in built environments (Das and Gailey, 2022; Tsokani et al., 2025; Zhu et al., 2023; Hu et al., 2025). For example, individuals who walk, jog, or cycle in green spaces report significantly improved psychological states and reduced stress, compared with performing the same activities in urban environments (Wicks et al., 2022; Bramwell et al., 2023). In one study, participants who engaged in activities such as hiking, gardening, or cycling showed significant increases in self-esteem and reductions in negative affect after the activity, whereas performing the same exercise in non-natural environments produced weaker effects (Gladwell et al., 2013). Similarly, walking in forests or parks has been found to improve emotional well-being and cognitive functioning more effectively than walking on urban streets (Berman et al., 2012; Ochiai et al., 2025). These findings consistently support the view that natural environments provide a favorable context for restoration and help accelerate recovery from stress and mental fatigue. Even brief bouts of green exercise—such as 10 to 15 min—have been associated with significant reductions in anxiety and tension (Mackay and Neill, 2010). Therefore, green exercise may serve as an effective stress-reduction strategy and a potential resource for coping with academic burnout. To date, research on green exercise has mainly focused on outcomes such as general mood, stress, or anxiety (Wicks et al., 2022; Lahart et al., 2019), while its specific association with academic burnout has not been thoroughly investigated. This makes the incorporation of green exercise into academic burnout research an important yet empirically underexplored direction.

Nature connectedness, also referred to as connectedness to nature or nature relatedness, is defined as a lasting sense of intimacy and attachment between the individual and the natural world (Mayer and Frantz, 2004). It reflects the extent to which people view nature as part of their identity and maintain close emotional and cognitive ties with it. Previous studies have shown that nature connectedness is an important psychological factor associated with well-being (Capaldi et al., 2014; Liao et al., 2025; Zhang et al., 2023). Individuals with stronger connections to nature typically report higher life satisfaction, happiness, and vitality, as well as lower anxiety and perceived stress (Zeng et al., 2025; Bakir-Demir et al., 2021). For example, people with high levels of nature connectedness often experience positive emotions and a sense of calm in natural environments, which contributes to improved overall mental health. A meta-analysis on nature connectedness found a reliable positive association between nature connectedness and indicators of well-being and positive affect (Capaldi et al., 2014). Moreover, survey research has shown that individuals with higher nature connectedness tend to exhibit fewer depressive symptoms and better psychological resilience (Ma et al., 2025). One explanation is that a close relationship with nature can provide meaning, tranquility, and a sense of social connection (albeit with the broader biotic community), helping individuals cope with everyday stress. Among student populations, strong nature connectedness may function as a psychological buffer: a student who feels “at home” in nature may recover more quickly from academic stress or seek comfort outside academic life by turning to natural environments. However, despite recent evidence of its protective role, research on the relationship between nature connectedness and academic burnout remains limited (Zhang et al., 2024). For example, a longitudinal study of Chinese adolescents found that students with higher baseline nature connectedness showed smaller increases in academic burnout, suggesting that nature connectedness may serve as a positive personal resource that reduces the risk of burnout (Zhang et al., 2024). From the perspective of COR, nature connectedness can be regarded as a relatively stable “psychological resource,” but how this resource interacts with specific nature-based behaviors (such as green exercise) to influence university students’ academic burnout still lacks systematic theoretical and empirical integration.

Although green exercise and nature connectedness are conceptually related, they are distinct to some extent. On the one hand, engaging in outdoor physical activity may naturally foster stronger connections with nature. Students who regularly jog in campus gardens or exercise in parks may gradually develop deeper emotional bonds with these natural settings—they may begin to notice and appreciate the surrounding scenery, seasons, and fresh air, thereby enhancing their nature connectedness. On the other hand, individuals with stronger nature connectedness may be more motivated to seek opportunities for outdoor activities because they inherently enjoy spending time in natural environments. Indeed, prior research suggests that this relationship is mutually reinforcing: recreational exposure to nature can increase individuals’ connection to nature, and those with higher nature connectedness are more likely to use nature for restoration (Baceviciene and Jankauskiene, 2022; Chang et al., 2024). In terms of mental health, both green exercise and nature connectedness are associated with reduced stress and improved well-being (Firnhaber et al., 2025; Gu et al., 2023), yet their combined effects on more severe outcomes such as academic burnout have not been fully explored. Theoretically, viewing green exercise as a “nature-based behavioral resource” and nature connectedness as a “nature-based psychological resource,” and examining their joint influence on academic burnout within a single model, can help us understand more precisely, within the framework of COR, SRT, and ART, how environmental behaviors and psychological resources relate to academic burnout. Practically, if this combination of behavioral and psychological resources is shown to be protective against academic burnout, it would provide a basis for universities to implement low-cost preventive interventions—such as building campus green spaces and designing physical education programs that incorporate nature exposure.

Although, in theory, green exercise and nature connectedness may help mitigate academic burnout, empirical research in this area remains scarce. Most studies on academic burnout have focused on factors such as study habits, personality traits, or social support (Ye et al., 2021; Kumyoung and Kumyoung, 2025), and have rarely addressed environmental and lifestyle factors such as exposure to nature. Therefore, several important questions remain unanswered: Is exercising in nature truly associated with lower levels of academic burnout among university students? Is feeling connected to nature related to academic exhaustion or cynicism? More importantly, can nature connectedness function as a mediator that helps explain why students who engage in green exercise experience less burnout? Clarifying these links may expand our understanding of burnout prevention by integrating perspectives from health psychology and environmental psychology. At the theoretical level, this study integrates COR, SRT, and ART into the context of academic burnout, attempting to extend environmental psychology theories that have traditionally been used to explain job burnout, general stress recovery, and attentional restoration, and apply them to the specific domain of university students’ academic burnout. At the practical level, this integration helps explore an operational, low-cost, and context-friendly intervention pathway—namely, alleviating academic burnout by promoting green exercise and enhancing nature connectedness.

**Figure 1:**
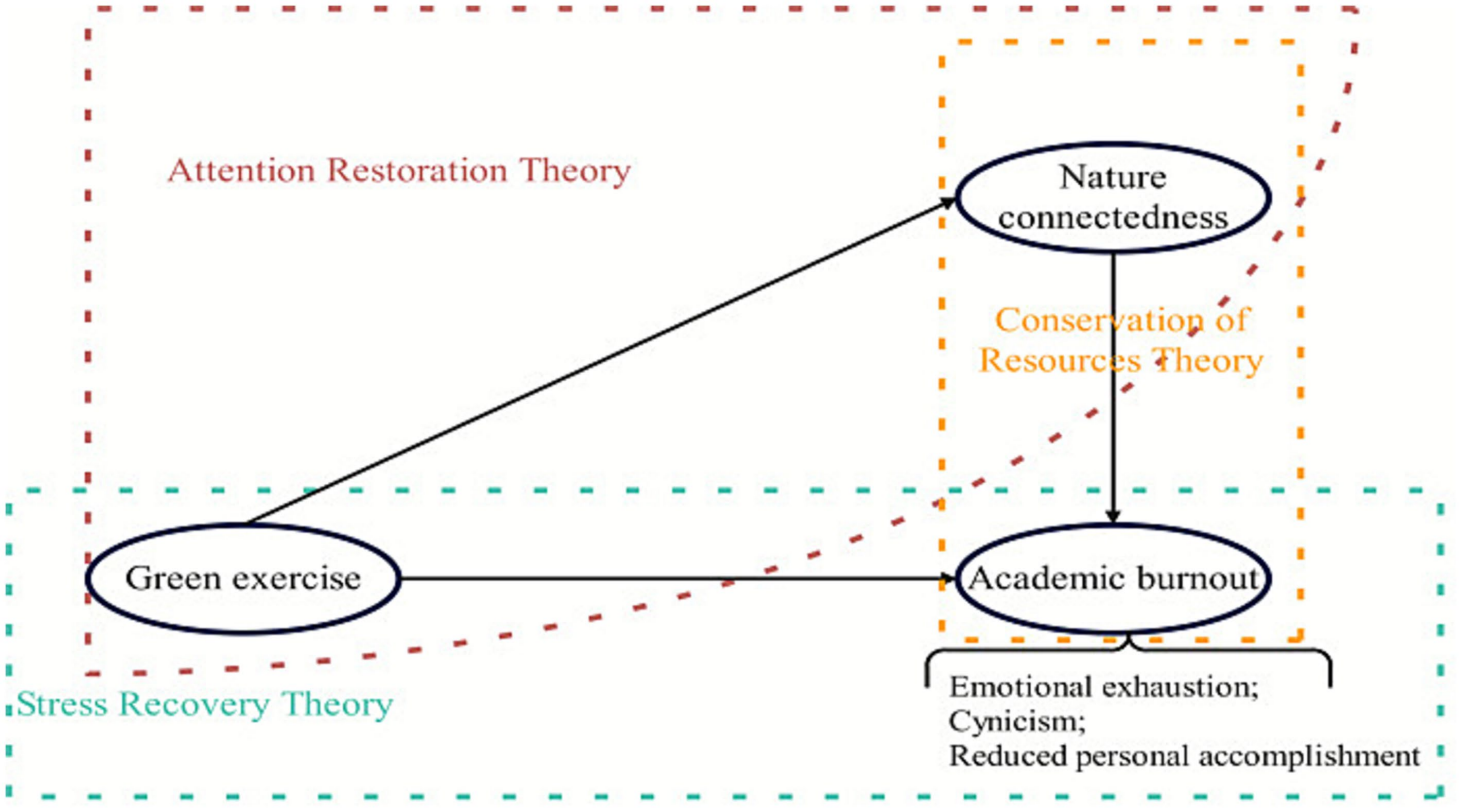
Theoretical hypothesized model of green exercise, nature connectedness, and academic burnout.

This study aims to fill these gaps by examining the relationships among green exercise, nature connectedness, and academic burnout in a large sample of Chinese university students. We propose a conceptual model in which green exercise and nature connectedness are positively associated with favorable burnout outcomes (i.e., lower emotional exhaustion and cynicism, and higher personal accomplishment), with nature connectedness potentially mediating the effect of green exercise on burnout. Figure 1 presents the hypothesized model: students who engage in more green exercise are expected to report higher nature connectedness, and higher nature connectedness is expected to be associated with lower academic burnout (i.e., lower emotional exhaustion and cynicism, and higher personal accomplishment). We also hypothesize that, even after accounting for the indirect effects via nature connectedness, there will remain a direct negative association between green exercise and academic burnout, reflecting the unique contribution of physical activity. Finally, we test the following hypotheses:

*H1*: Green exercise will be positively associated with nature connectedness.

*H2*: Green exercise will be negatively associated with academic burnout.

*H3*: Nature connectedness will mediate the relationship between green exercise and academic burnout, such that students with higher engagement in green exercise experience lower academic burnout in part through greater nature connectedness.

By testing these hypotheses with structural equation modeling (SEM), this study aims to provide novel insights into how lifestyle and environmental connectedness factors intertwine with academic well-being. The findings will contribute to the theoretical integration of environmental psychology and educational psychology and suggest practical strategies for reducing student burnout through nature-based approaches.

## 2 Method

### 2.1 Participants

This study adopted a stratified random sampling method, with participants drawn from first- to fourth-year undergraduate students at 10 universities located in Sichuan, Chongqing, Guizhou, and Yunnan, China. The gender distribution of the sample was approximately balanced (55% female), and participants’ ages ranged from 18 to 24 years (M ≈ 20.3, SD ≈ 1.4). In the specific sampling procedure, universities and grade levels were used as the main stratification variables. Within each university, classes or teaching units of first- to fourth-year students served as the basic sampling units, and questionnaires were distributed to multiple classes with the assistance of local instructors to ensure that students from different grades and institutions were adequately represented, thereby forming a cross-institutional stratified random sample. In order to comply with the standard empirical rule in social science research, the recommended sample size is typically 10 to 15 times the total number of questionnaire items (Jeffrey and Hoogland, 1998). The measurement instruments used in this study included the Green Exercise Scale (5 items), the Nature Connectedness Scale (14 items), and the Academic Burnout Scale (14 items), comprising a total of 33 items; therefore, based on this rule, the required sample size was estimated to range from 330 to 495 participants. Data were collected from 15 March 2025 to 30 September 2025, during which questionnaires were distributed to approximately 1,200 students; these 1,200 questionnaires correspond to the number of students initially invited to participate in the survey. During questionnaire return and data cleaning, we excluded invalid responses due to missing data, inconsistent answers, or fixed response patterns, and ultimately obtained 1,022 valid questionnaires for analysis, yielding an effective response rate of 85.17%. In this study, all successfully submitted questionnaires were regarded as participants who “started the survey” and were included in the data analysis; however, because data collection combined both the Wenjuanxing online platform and in-class paper administration, we did not separately record the number of students who opened but did not complete the online questionnaire, and thus could not further distinguish those who began but did not finish the survey. Recruitment was carried out by contacting physical education instructors or class advisors at each institution, who introduced the study purpose and participation procedure to the students in their classes and assisted in distributing the questionnaires. Announcements were made through the distribution of paper questionnaires in class as well as via the Wenjuanxing online platform. Some students received and completed paper questionnaires during class time, which were collected on site by course instructors or research assistants, while others completed the survey online using their mobile phones or computers by accessing the Wenjuanxing link, which was shared in the classes overseen by physical education instructors or class advisors. Overall, the study adopted a data collection strategy that relied primarily on online questionnaires supplemented by in-class paper questionnaires across the 10 universities, without using a university-wide academic affairs system for mass mailing; instead, recruitment and distribution were conducted at the class level through instructors and class advisors. Participation was entirely voluntary and anonymous. Before taking part, all participants received verbal informed consent information regarding the study purpose, estimated completion time, and confidentiality principles, and they began the survey only after agreeing to participate. Non-participation or withdrawal at any time had no consequences for students’ coursework or grades. This study was approved by the Ethics Committee of Chengdu Sport University (approval number: CTYLL2024017).

### 2.2 Measurement tools

#### 2.2.1 Green exercise

Green exercise was assessed using a five-item scale developed by Wood et al. (2019). Items measured the frequency of engaging in physical activities in natural environments (e.g., “I often exercise in parks or other green spaces,” “I choose outdoor environments such as campus gardens or forests to exercise whenever possible”). Responses were rated on a 5-point Likert scale (1 = strongly disagree, 5 = strongly agree). Higher scores indicated greater participation in green exercise. This scale showed good reliability in the present study (Cronbach’s *α* = 0.860).

#### 2.2.2 Nature connection

Nature connection was measured using the 14-item Connectedness to Nature Scale (CNS) developed by Li and Wu (2016). Items assessed emotional and cognitive bonds with the natural world (e.g., “I feel like I am part of the network of life”). Each item was rated on a 5-point Likert scale (1 = strongly disagree, 5 = strongly agree). Higher scores reflected stronger nature connectedness. Internal consistency was excellent (Cronbach’s α = 0.917).

#### 2.2.3 Academic burnout

Academic burnout was assessed using the 14-item scale developed by Ni et al. (2009), which captures three dimensions: emotional exhaustion (4 items), cynicism (4 items), and reduced personal accomplishment (6 items). Example items included “I feel emotionally drained by my academic work” (emotional exhaustion), “I lose interest in my courses and start questioning their value” (cynicism), and “Recently, I feel that my academic achievements are not fulfilling” (reduced personal accomplishment). Items were rated on a 5-point frequency scale (1 = never, 5 = almost always). Higher scores on emotional exhaustion and cynicism, as well as reversed scores on reduced personal accomplishment, indicated greater burnout. Subscale reliabilities were satisfactory (EE: α = 0.807, CY: α = 0.818, RPA: α = 0.867).

In addition to the primary measurement tools mentioned above, the questionnaire also collected basic demographic information (age, gender, year level) and inquired about general health and study load, which were used for sample description and exploratory control checks. However, no significant effects of demographic characteristics on key variables were observed, so these were not included as covariates in the final model for simplicity.

### 2.3 Procedure

The survey was administered from March 15, 2025, to September 30, 2025. Data collection was primarily conducted through two methods: First, using the Wenjuanxing survey platform, the questionnaire was distributed to students as a link for them to complete online; second, by contacting sports teachers and class advisors to administer paper-based surveys in person (with an average completion time of about 15 min). To minimize potential common method bias, several procedural remedies were implemented: Participants were assured of anonymity and informed that there were no right or wrong answers. The order of some sections was balanced (for example, academic burnout items were separated from green exercise and nature connection items by filler questions). All items were presented in Chinese. For the scales originally in English, we either used published Chinese translations or translated the items following a standard forward-backward translation procedure to ensure linguistic accuracy. Prior to the main study, the questionnaire was pilot-tested with a group (N = 30) to ensure the clarity of the Chinese items and the proper functioning of the online form. Based on their feedback, wording adjustments were made. After completing the survey, participants were informed about the purpose of the research. No identifying information was collected, and participants were offered the option to enter a raffle via email to win small prizes as compensation, while maintaining the anonymity of the survey responses.

### 2.4 Data analysis

All statistical analyses were performed using *SPSS 27.0* and *Mplus 8.3*. The analytical procedures comprised four steps: assessment of common method bias, evaluation of the measurement model, correlation analysis, and structural equation modeling with mediation testing.

First, descriptive statistics were calculated for all study variables, including means and standard deviations. Reliability was assessed using Cronbach’s *α*, and the psychometric properties of the latent constructs were further evaluated through composite reliability (CR) and average variance extracted (AVE).

Second, confirmatory factor analyses (CFA) were conducted to examine the construct validity of the measurement model. Competing models were compared, including a single-factor model, the hypothesized five-factor model (GE, NC, EE, CY, RPA as separate latent constructs), and a six-factor model that incorporated an additional method factor. Model fit was evaluated with multiple indices: chi-square statistic and its degrees of freedom (χ^2^/df), Comparative Fit Index (CFI), Tucker–Lewis Index (TLI), Root Mean Square Error of Approximation (RMSEA) with 90% confidence interval, and Standardized Root Mean Square Residual (SRMR). Thresholds of CFI and TLI ≥ 0.90, RMSEA ≤ 0.08, and SRMR ≤ 0.08 were used as benchmarks of acceptable model fit (Wen and Ye, 2014).

Third, Pearson correlation coefficients were computed to examine the bivariate associations among GE, NC, and the three dimensions of Academic Burnout (EE, CY, RPA). Discriminant validity was further assessed by comparing the square roots of AVE with the inter-construct correlations (Tang and Wen, 2020).

Finally, structural equation modeling (SEM) with maximum likelihood estimation was used to estimate the hypothesized relationships. In the structural model, GE and NC were modeled as latent constructs, and EE, CY, and RPA were modeled as three distinct latent constructs representing academic burnout. Both direct and indirect effects of GE on burnout were estimated, with NC specified as the mediating variable. The significance of indirect effects was tested using bias-corrected bootstrapping with 5,000 resamples, and 95% confidence intervals were generated. The proportion of total effects accounted for by indirect effects was also calculated. All statistical tests were two-tailed, with significance set at *p* < 0.05 (Wen et al., 2004).

## 3 Result

### 3.1 Common method bias assessment

To evaluate the potential influence of common method bias (CMB), both Harman’s single-factor test and confirmatory factor analysis (CFA) of alternative measurement models were performed. The exploratory factor analysis revealed that six factors together accounted for 60.81% of the total variance, with the first factor explaining 30.85%. Since the variance explained by the first factor did not exceed the critical threshold of 40%, serious common method bias was unlikely to threaten the validity of the results.

To further assess this issue, three CFA models were compared: (a) a single-factor model in which all measurement items loaded onto one general factor, (b) a five-factor model corresponding to the theoretical constructs (GE, NC, EE, CY, RPA), and (c) a six-factor model that included the theoretical factors plus an additional latent method factor. As shown in Table 1, the hypothesized five-factor model exhibited a significantly better fit than the single-factor model (χ^2^/df = 1.685, CFI = 0.978, TLI = 0.976, SRMR = 0.033, RMSEA = 0.026 vs. χ^2^/df = 13.191, CFI = 0.596, TLI = 0.569, SRMR = 0.113, RMSEA = 0.109). The six-factor model, which included the common method factor, did not provide additional improvement in fit indices (χ^2^/df = 1.688, CFI = 0.978, TLI = 0.976, SRMR = 0.033, RMSEA = 0.026). Together, these results indicate that common method bias was not a serious concern in the present study.

**Table 1:** Fit indices for alternative measurement models.

Model	χ^2^/df	CFI	TLI	SRMR	RMSEA
One-factor	13.191	0.596	0.569	0.113	0.109 (0.107–0.112)
Five-factor	1.685	0.978	0.976	0.033	0.026 (0.023–0.029)
Six-factor	1.688	0.978	0.976	0.033	0.026 (0.023–0.029)

One-factor model = all items loading on a single general factor; Five-factor model = each variable loading on its theoretical factor (GE, NC, EE, CY, RPA); Six-factor model = theoretical five factors plus an additional method factor.

### 3.2 Measurement model and descriptive statistics

Descriptive statistics, internal consistency reliability, and confirmatory factor analysis (CFA) indices for the main study variables are presented in Table 2. The mean scores for Green Exercise (GE) and Nature Connectedness (NC) were 3.37 (SD = 0.81) and 3.25 (SD = 0.64), respectively, indicating moderate engagement in green exercise and a comparable sense of connection to nature among participants. The three dimensions of Academic Burnout—Emotional Exhaustion (EE), Cynicism (CY), and Reduced Personal Accomplishment (RPA)—showed mean values between 3.05 and 3.17, suggesting moderate burnout levels overall.

**Table 2:** Descriptive statistics, internal consistency reliability, and fit indices for confirmatory factor analysis (CFA) of key variables.

Variable	Mean	SD	*α*	Factor loading	CR	AVE
GE	3.37	0.81	0.860	0.697–0.854	0.862	0.557
NC	3.25	0.64	0.917	0.424–0.730	0.920	0.459
EE	3.17	0.78	0.807	0.684–0.731	0.807	0.511
CY	3.05	0.82	0.818	0.716–0.745	0.819	0.531
RPA	3.06	0.80	0.867	0.708–0.750	0.867	0.521

GE = Green exercise; NC = Nature connectedness; EE = Emotional exhaustion; CY = Cynicism; RPA = Reduced personal accomplishment.

All constructs demonstrated satisfactory internal consistency reliability, with Cronbach’s *α* coefficients ranging from 0.807 (EE) to 0.917 (NC). Composite reliability (CR) values ranged between 0.807 and 0.920, all exceeding the recommended threshold of 0.70. Average variance extracted (AVE) values ranged from 0.459 to 0.557. Although the AVE for NC (0.459) was slightly below the conventional criterion of 0.50, its high CR (0.920) and acceptable factor loadings (0.424–0.730) supported its convergent validity. Factor loadings for all constructs ranged from 0.424 to 0.854, with most items above 0.60, providing additional evidence of adequate measurement properties. Overall, the results indicate that the measurement model exhibited satisfactory psychometric properties and was suitable for subsequent structural analyses.

### 3.3 Correlation analysis

Pearson correlation coefficients among the key study variables are presented in Table 3, with the square roots of the average variance extracted (AVE) shown on the diagonal. Green Exercise (GE) was positively correlated with Nature Connectedness (NC; *r* = 0.320, *p* < 0.001), indicating that students who engaged more in green exercise tended to report higher levels of nature connectedness. GE was also negatively correlated with the three dimensions of Academic Burnout: Emotional Exhaustion (EE; *r* = −0.284, *p* < 0.001), Cynicism (CY; *r* = −0.256, *p* < 0.001), and Reduced Personal Accomplishment (RPA; *r* = −0.299, *p* < 0.001). Similarly, NC showed significant negative correlations with EE (*r* = −0.394, *p* < 0.001), CY (*r* = −0.380, *p* < 0.001), and RPA (*r* = −0.411, *p* < 0.001). These correlation results provided preliminary support for H1 and H2, showing that green exercise was positively related to nature connectedness and negatively related to academic burnout dimensions.

**Table 3:** Correlations and discriminant validity among key variables.

Variable	GE	NC	EE	CY	RPA
GE	0.746				
NC	0.320^***^	0.677			
EE	−0.284^***^	−0.394^***^	0.715		
CY	−0.256^***^	−0.380^***^	0.538^***^	0.729	
RPA	−0.299^***^	−0.411^***^	0.524^***^	0.532^***^	0.722

^***^p<0.001. GE = Green exercise; NC = Nature connectedness; EE = Emotional exhaustion; CY = Cynicism; RPA = Reduced personal accomplishment. Values on the diagonal represent the square roots of AVE.

The discriminant validity of the constructs was further supported, as the square roots of AVE (diagonal values ranging from 0.677 to 0.746) were greater than the corresponding inter-construct correlations, confirming that the latent variables were empirically distinct.

### 3.4 Structural equation modeling

Structural equation modeling (SEM) was performed to examine the relationships among Green Exercise (GE), Nature Connectedness (NC), and the three dimensions of Academic Burnout (Emotional Exhaustion, Cynicism, and Reduced Personal Accomplishment). The structural model demonstrated excellent overall fit to the data, with all indices exceeding conventional thresholds (χ^2^/df = 1.686, CFI = 0.978, TLI = 0.976, SRMR = 0.032, RMSEA = 0.026, 90% CI = 0.023–0.029). The fit indices are summarized in Table 4.

**Table 4:** Model fit indices of the hypothesized structural model.

Model fit	χ*2*/*df*	CFI	TLI	SRMR	RMSEA (90%CI)
Model	1.686	0.978	0.976	0.032	0.026 (0.023–0.029)

**Figure 2:**
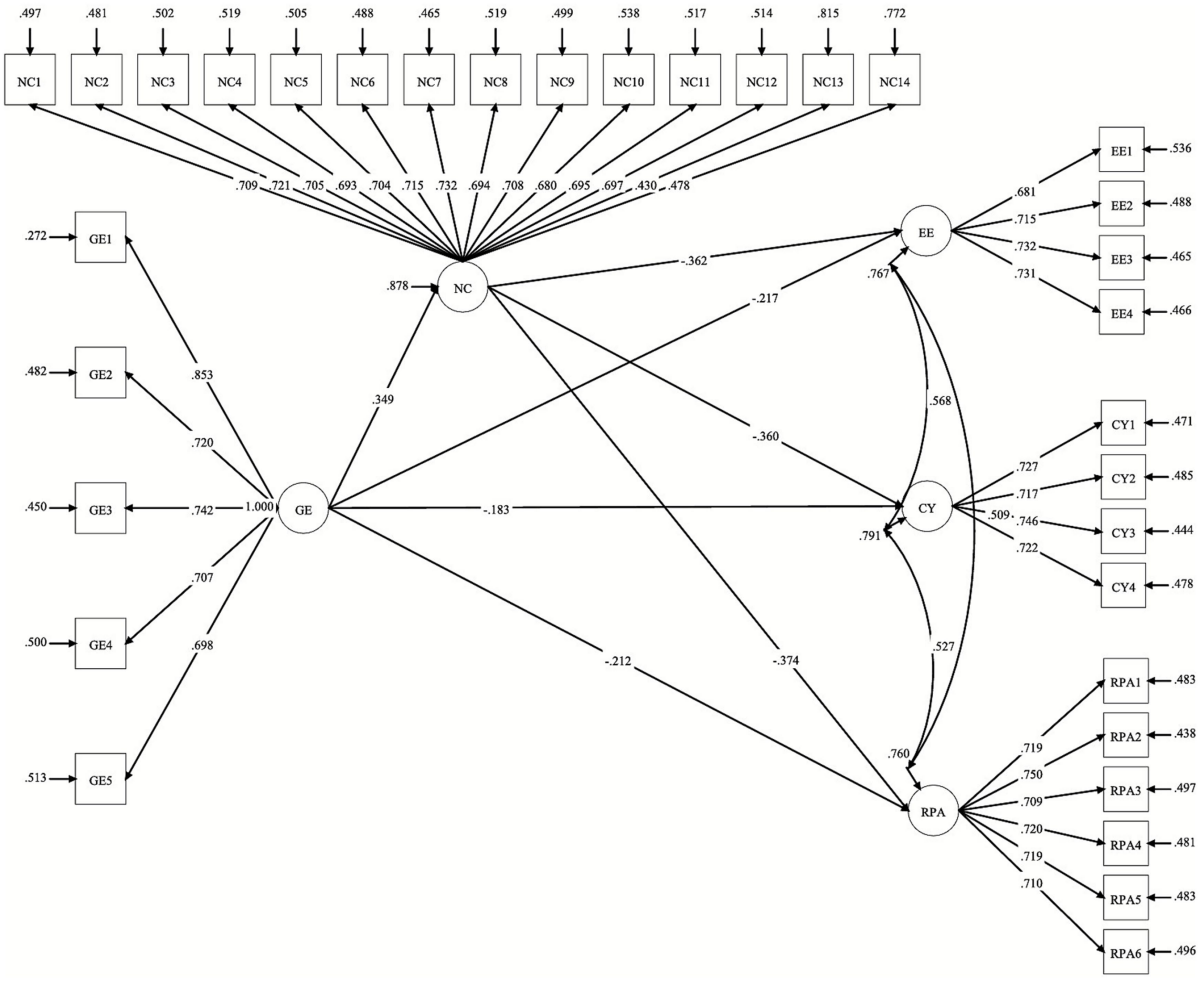
Standardized path coefficients of the structural model.

The standardized path coefficients are presented in Figure 2. All estimated paths were statistically significant at the 0.01 level, indicating that GE positively predicted NC, and both GE and NC exerted significant negative effects on the three burnout dimensions. The SEM results offered direct evidence for H1 and H2, confirming that green exercise positively predicted nature connectedness and negatively predicted academic burnout.

### 3.5 Mediation analysis

Bias-corrected bootstrapping with 5,000 resamples was conducted to assess the indirect effects of Green Exercise (GE) on the three dimensions of Academic Burnout through Nature Connectedness (NC). The total, direct, and indirect effects are presented in Table 5.

**Table 5:** Total, direct and indirect effects in the model.

Path	*β*	Boot SE	*p*	Boot LLCI	Boot ULCI	Ratio
GE→EE						
Direct effect	−0.217	0.036	< 0.001	−0.226	−0.113	63.27%
Indirect effects	−0.126	0.017	< 0.001	−0.129	−0.074	36.73%
Total effect	−0.343	0.034	< 0.001	−0.322	−0.212	100%
GE→CY						
Direct effect	−0.183	0.037	< 0.001	−0.227	−0.097	59.22%
Indirect effects	−0.126	0.016	< 0.001	−0.147	−0.086	40.78%
Total effect	−0.309	0.036	< 0.001	−0.337	−0.209	100%
GE→RPA						
Direct effect	−0.212	0.035	< 0.001	−0.250	−0.124	61.81%
Indirect effects	−0.131	0.017	< 0.001	−0.150	−0.088	38.19%
Total effect	−0.343	0.033	< 0.001	−0.365	−0.238	100%

GE = Green exercise; NC = Nature connectedness; EE = Emotional exhaustion; CY = Cynicism; RPA = Reduced personal accomplishment. Boot LLCI, the lower bound of the 95% confidence interval. Boot ULCI, the upper limit of the 95% confidence interval (Percentile Bootstrap Method with Bias Correction). The Bootstrap sample size is set at 5000.

For Emotional Exhaustion (EE), GE exerted a significant direct effect (*β* = −0.217, SE = 0.036, *p* < 0.001) and a significant indirect effect via NC (*β* = −0.126, SE = 0.017, *p* < 0.001, 95% CI [−0.129, −0.074]). The total effect was −0.343, with the indirect pathway accounting for 36.73% of the overall effect.

For Cynicism (CY), GE showed a significant direct effect (*β* = −0.183, SE = 0.037, *p* < 0.001) and a significant indirect effect through NC (*β* = −0.126, SE = 0.016, *p* < 0.001, 95% CI [−0.147, −0.086]). The total effect was −0.309, with the indirect effect contributing 40.78% of the total.

For Reduced Personal Accomplishment (RPA), GE had a significant direct effect (*β* = −0.212, SE = 0.035, *p* < 0.001) and a significant indirect effect mediated by NC (*β* = −0.131, SE = 0.017, *p* < 0.001, 95% CI [−0.150, −0.088]). The total effect was −0.343, with the indirect effect explaining 38.19% of the overall association.

These results indicate that NC partially accounted for the associations between GE and all three burnout dimensions, with both direct and indirect pathways being statistically significant. These findings supported H3, indicating that nature connectedness partially mediated the associations between green exercise and the three dimensions of academic burnout.

## 4 Discussion

### 4.1 Green exercise and reduction in academic burnout

Focusing on the first research question—whether green exercise is associated with lower academic burnout—the results indicate that participating in physical activities in natural environments is significantly related to lower levels of academic burnout among university students. This finding extends the established mental health benefits of physical exercise and nature exposure to the academic domain. Within stress-reduction frameworks, exercise promotes emotional recovery by enhancing physiological regulation, improving sleep, and helping individuals psychologically detach from stressors (Bernstein and McNally, 2018; Korkutata et al., 2025), while restorative environment theory emphasizes that natural environments play a crucial role in replenishing depleted cognitive and emotional resources (Ohly et al., 2016). The combination of these mechanisms provides a plausible explanation for how green exercise can directly protect students’ mental and physical health.

Notably, the analyses show that even after accounting for the mediating effect of nature connectedness, green exercise still maintains a significant direct association with reduced academic burnout. This suggests that the benefits of outdoor physical activity cannot be fully explained by attitudinal or cognitive changes related to nature; rather, the activity itself, conducted in a restorative environment, constitutes a substantive resource for coping with academic stress. This evidence is consistent with Conservation of Resources (COR) theory, which emphasizes the necessity of resource replenishment for preventing burnout (Demerouti, 2025).

The effects of green exercise were observed across all three dimensions of academic burnout. The strongest direct association was with emotional exhaustion, which aligns with previous research suggesting that emotional exhaustion is the core component of burnout and is particularly sensitive to stress-reduction interventions. Regular physical activity in natural environments appears to alleviate fatigue and stress responses, thereby reducing emotional exhaustion more effectively than the other dimensions. The negative association with cynicism indicates that students who engage in green exercise may maintain a more positive attitude toward their academic tasks, possibly because periodic exposure to natural environments helps prevent the accumulation of negative attitudes and detachment toward learning. The association with personal accomplishment suggests that these students maintain a stronger sense of efficacy and competence in their academic work. This may be attributed to the role of physical activity in enhancing self-esteem and perceived control, as well as to the emotional and cognitive benefits of natural environments in promoting psychological balance and broadening perspectives (Martín-Rodríguez et al., 2024).

Taken together, with respect to the first research question, these findings highlight green exercise as a multifaceted protective factor: it operates through both direct and indirect pathways, reducing emotional exhaustion, attenuating cynical attitudes, and enhancing personal accomplishment, thereby strengthening students’ resilience in the face of academic demands.

### 4.2 Nature connectedness as a buffering factor

With regard to the second research question, the results show a significant negative association between nature connectedness and academic burnout, indicating that nature connectedness is an important psychological resource that can buffer the impact of chronic academic stress. This finding is consistent with recent studies suggesting that nature connectedness can be viewed as a relatively stable individual trait that enhances coping and resilience, thus providing protection under stressful conditions (Ma et al., 2025). Individuals who feel more closely connected to nature may possess stronger psychological restorative capacities and emotion regulation abilities, thereby reducing their vulnerability to prolonged stress responses that underlie burnout.

The negative association between nature connectedness and reduced personal accomplishment was particularly pronounced. This pattern suggests that feeling closely connected to the natural environment may help students maintain a stronger sense of efficacy and competence in the academic domain. From the perspectives of existential psychology and positive psychology, nature connectedness can provide individuals with a sense of meaning, enhance self-confidence, and expand self-identity beyond academic performance (Howell et al., 2013). The broaden-and-build theory of positive emotions offers a complementary perspective: positive emotions elicited by a sense of oneness with nature can broaden individuals’ cognitive flexibility and psychological resources, thereby enhancing their resilience to academic stress (Fredrickson, 2001).

Beyond psychological mechanisms, nature connectedness may also exert physiological and behavioral influences. Prior research has shown that higher levels of nature connectedness are associated with lower anxiety, higher vitality, and better stress recovery (Chang et al., 2024). These benefits can be obtained not only through direct contact with natural environments, but also through an internalized sense of affinity with nature that can be activated in everyday contexts. From the perspective of the biophilia hypothesis, humans have an inherent need to affiliate with nature, and the satisfaction of this need appears to help stabilize emotional states and facilitate stress recovery, thereby preventing burnout to some extent (von Lindern et al., 2017).

This study adds to the limited empirical literature on this relationship in higher education contexts. Recent longitudinal research on adolescents has found that stronger nature connectedness predicts lower levels of learning burnout (Zhang et al., 2024). Extending these findings to a university student population, the present study suggests, in relation to the second research question, that nature connectedness may be a key yet relatively overlooked factor in promoting academic well-being across different developmental stages.

### 4.3 Mediation via nature connectedness

Addressing the third research question—whether nature connectedness mediates the relationship between green exercise and academic burnout—the mediation analysis showed that approximately one-third to two-fifths of the effect of green exercise on academic burnout was transmitted indirectly through nature connectedness. This indicates that part of green exercise’s protective effect operates by strengthening students’ psychological connection to the natural environment, which in turn helps them cope with academic stress in a more adaptive manner. These findings support the view that regular participation in outdoor physical activity not only provides immediate stress relief, but also fosters a deeper sense of nature connectedness that functions as a long-term resilience resource.

The observation of partial mediation is theoretically meaningful. It suggests that nature connectedness is an important, but not exclusive, pathway linking green exercise to reduced academic burnout. This mediation model is consistent with restorative environment theory and the broaden-and-build theory of positive emotions, both of which emphasize that emotional and cognitive connections with natural environments can broaden psychological resources and facilitate stress recovery (Baceviciene and Jankauskiene, 2022; Fredrickson, 2001). At the same time, the persistence of direct effects indicates that mechanisms other than nature connectedness also play a role. It is well established that physical exercise can regulate neurobiological processes (e.g., lowering cortisol levels, increasing endorphins and brain-derived neurotrophic factor) and improve sleep quality and circadian rhythm balance, all of which can independently contribute to stress reduction (De Nys et al., 2022; Hossain et al., 2024). Furthermore, theories of stress recovery and psychological detachment suggest that outdoor exercise can provide psychological distance from academic demands, enabling students to temporarily “step away” from study-related pressures and preventing the continuous accumulation of stress, even if their subjective attitudes toward nature are not particularly positive (Ohly et al., 2016).

Therefore, the coexistence of indirect and direct effects underscores the multidimensional nature of green exercise as a protective factor. With respect to the third research question, nature connectedness can be seen as an important bridge between green exercise and reduced academic burnout, while at the same time, other physiological, behavioral, and social mechanisms also play crucial roles. This multifactorial model suggests that, in order to understand the mental health benefits of green exercise, it is necessary to consider both environmental dimensions and the characteristics of the activity itself.

### 4.4 Theoretical integration and international applicability

Overall, the findings of this study can be interpreted through the lenses of Conservation of Resources (COR) theory, Stress Reduction Theory (SRT), and Attention Restoration Theory (ART). First, COR theory posits that stress and burnout arise when individuals experience actual or threatened loss of key resources (such as energy, optimism, or self-efficacy) or when resource investment fails to yield expected returns (Hobfoll, 1989). Green exercise can be conceptualized as a “resource-enhancing” activity: it promotes physical health and vitality, provides opportunities for recovery, and generates positive emotional experiences. At the same time, nature connectedness can be viewed as a psychosocial resource—similar to social support or a sense of meaning—that enhances individuals’ coping capacity. From this perspective, green exercise and nature connectedness together form what COR theory describes as a “resource caravan”: a set of interrelated resources that accumulate and reinforce one another (Hobfoll, 1989). The evidence from this study suggests that students who engage in green exercise not only replenish depleted resources through the activity itself, but also build enduring psychological capital through stronger nature connectedness, thereby breaking the vicious cycle of resource loss and reducing their risk of burnout.

Second, these findings can also be understood within stress and coping frameworks. Whether individuals possess effective strategies to manage external demands and regulate emotions determines how successful their coping efforts will be. Green exercise can be seen as a proactive coping strategy that provides both physiological stress relief and psychological support, whereas nature connectedness contributes to more adaptive cognitive appraisal and emotion regulation processes. Students with higher levels of nature connectedness may be more inclined to use nature as a coping resource: actively seeking restorative environments under stress, or drawing comfort from an internalized sense of connection with nature in everyday life. This “dual pathway”—behavioral engagement through green exercise and attitudinal/cognitive orientation through nature connectedness—suggests that the natural environment functions both as an external coping context and as an internalized coping asset. At the same time, from the perspectives of SRT and ART, green exercise represents a behavioral channel through which emotional and attentional restoration is achieved in natural settings, whereas nature connectedness reflects a sustained emotional and cognitive openness and commitment to nature. Together, these elements constitute a complete process from resource acquisition to resource internalization and accumulation.

In summary, by integrating COR theory, SRT/ART, and stress–coping perspectives, the present findings emphasize that green exercise and nature connectedness are interdependent resources operating at both behavioral and psychological levels. These resources help to strengthen resilience, facilitate recovery, and slow down the accumulation of stress, thereby reducing the occurrence of academic burnout among university students. Although this study was conducted in the context of Chinese higher education, the underlying theoretical mechanisms are likely to possess a certain degree of cross-cultural applicability. On the one hand, heavy academic workloads, competitive evaluation systems, and limited leisure time are structural stressors shared by many higher education systems worldwide, suggesting that conceptualizing nature as a “restorative resource” and “coping resource” may also be relevant in other contexts. On the other hand, differences across countries in campus green-space design, physical education curricula, and students’ opportunities for contact with nature may influence the specific forms and strength of the effects of green exercise and nature connectedness. Thus, the findings of this study offer an environmental psychology perspective that can inform comparison and reflection in international higher education: universities in different countries can, within their local cultural and campus environmental conditions, explore how optimizing access to natural environments and physical activity opportunities might help mitigate students’ academic burnout. Future research should conduct cross-cultural or multi-country replication studies under different cultural and institutional contexts to test the applicability of the present model in broader international higher education settings and to further enrich the dialogue between environmental psychology and educational psychology in the field of academic burnout.

### 4.5 Limitations and future directions

Several limitations should be considered when interpreting the present findings.

First, the cross-sectional design restricts causal inference. Although the mediation analyses were conducted on theoretically grounded pathways, longitudinal or experimental studies are required to determine whether green exercise and nature connectedness actively reduce academic burnout over time.

Second, the exclusive reliance on self-reported data raises concerns about common method bias and subjective inaccuracies. Although procedural and statistical techniques were employed to minimize this issue, self-reports of exercise behavior, nature connectedness, and burnout symptoms remain susceptible to recall error and social desirability. Future research would benefit from incorporating multi-method assessments, such as accelerometer-based measures of outdoor activity, geolocation data on time spent in green spaces, physiological indicators of stress (e.g., cortisol), or behavioral ratings from peers and educators.

Third, the operationalization of green exercise and nature connectedness may not have fully captured the complexity of these constructs. Green exercise was assessed primarily in terms of frequency and subjective engagement, without accounting for exercise intensity, type, or social context, each of which may differentially influence outcomes. Nature connectedness was treated as a unidimensional construct, whereas cognitive, affective, and experiential facets may have distinct associations with well-being. Future research could examine whether particular subdimensions of connectedness are more protective against burnout.

Fourth, the sample was composed of Chinese university students. This context is characterized by both rich cultural traditions emphasizing harmony with nature and highly competitive academic environments. The combination of accessible campus green spaces and academic pressure may have shaped the observed relationships in specific ways. Caution should be exercised in generalizing the findings to other cultural or educational settings, though the underlying associations are expected to have cross-cultural relevance given the universal physiological and psychological benefits of exercise and natural environments.

Finally, alternative explanatory mechanisms cannot be ruled out. The observed association between green exercise and lower burnout may be partially attributable to third variables such as general health status, personality traits, or lifestyle factors. In addition, while nature connectedness was identified as a key mediator, other plausible mechanisms include improved mood, enhanced sleep quality, or social interaction during outdoor activity. Future research employing experimental or longitudinal designs should incorporate multiple mediators to provide a more comprehensive account of how green exercise influences academic well-being.

## 5 Practical implications

The present study’s outcomes can inform actionable strategies for students, educators, and university administrators to help prevent or reduce academic burnout. Below, we outline several practical implications and recommendations based on our findings:

### 5.1 Encourage regular green exercise

Universities should promote opportunities for students to engage in physical activities outdoors. This could include organizing group exercise sessions like morning jogs, yoga classes, or tai chi in campus green areas. Physical education programs or student clubs could incorporate nature-based activities (hiking trips, cycling outings, outdoor sports) as part of their offerings. The key is to make it easy and appealing for students to be active in natural settings. Our results suggest that even moderate, routine activities (e.g., a 30-min walk in a park a few times a week) could help students manage stress and protect against burnout. Universities might develop campaigns or challenges (e.g., “Green Exercise Challenge: 1000 steps in the campus garden each day”) to nudge students toward these habits.

### 5.2 Leverage campus green spaces

The campus environment itself can be a tool for student well-being. Institutions should invest in maintaining and enhancing green spaces like parks, gardens, tree-lined paths, and open lawns on campus. These areas serve as convenient venues for students to take restorative breaks or exercise. Ensuring such spaces are safe, attractive, and accessible is important – for instance, adequate lighting for evening walks, benches for relaxation, and perhaps even outdoor exercise equipment in some parks. Some universities could create “outdoor study areas” or gazebos in gardens, allowing students to combine nature exposure with work breaks. The concept of a “green campus” not only aids sustainability but, as our study indicates, can directly contribute to reducing student burnout by embedding nature into daily student life.

### 5.3 Integrate nature into stress-management programs

University counseling centers and wellness programs might incorporate nature-based activities into their stress-reduction workshops or therapy offerings. For example, counselors could lead mindfulness meditation sessions held outdoors (capitalizing on natural sights and sounds to facilitate mindfulness), or organize nature therapy groups where students discuss stress while on a guided nature walk. Many students experiencing burnout may not seek formal counseling due to stigma or busy schedules, but framing an activity as a “nature outing for stress relief” might attract participation from those who would not otherwise engage in support services. Additionally, educational seminars can teach students about the mental health benefits of spending time in nature and how to build such practices into their routine (e.g., taking a short walk in a green area between classes to clear one’s head). Simply raising awareness – for instance, sharing research (like “10 min in nature can reduce stress hormones”) – could motivate students to utilize nearby nature as a self-care resource.

### 5.4 Foster nature connectedness

Beyond promoting physical activity, universities and educators can help students develop a deeper connection to nature, which our findings suggest is a buffer against burnout. This can be achieved through curricular and extracurricular means. For example, coursework or projects that involve nature (such as biology or environmental science field trips, outdoor art or photography assignments, or readings/reflections on nature in literature or philosophy classes) can prompt students to engage with and reflect on the natural world. Extracurricularly, institutions might support clubs like outdoor adventure clubs, gardening clubs, or sustainability groups, all of which inherently boost time spent in nature and appreciation for it. Even one-off events – tree planting drives, campus nature scavenger hunts, or “bio-blitz” days where students catalog campus biodiversity – can strengthen students’ sense of connectedness with their environment. Campuses could also install signage about local flora/fauna or create small nature trails with descriptions, subtly educating and connecting students to nature around them. The more students see themselves as part of nature, the more likely they are to seek it out in times of stress and gain comfort from it.

### 5.5 Design academics with breaks and nature in mind

Academic schedules could be structured to allow short restorative breaks that encourage stepping outside. For instance, professors might be encouraged to give a 5-min break during long lectures and, weather permitting, suggest students get a breath of fresh air or a quick walk instead of scrolling on their phones. Universities could experiment with holding certain classes or discussion sections outdoors when feasible, which might both improve attention (per some studies) and provide a change of scenery that reduces monotony and stress. During high-pressure periods like exams, institutions could provide “nature rooms” or relaxation spaces filled with plants, natural light, and calming nature sounds/videos as a respite for students. Some universities have already tried “pet therapy” with animals on campus during exams – similarly, nature therapy stations could be set up (even a small indoor garden or a quiet courtyard) for students to decompress.

### 5.6 Policy and infrastructure support

At an administrative level, recognizing green exercise and nature connectedness as components of student mental health can influence campus planning and policies. For example, investment in outdoor recreational facilities (running tracks, bike paths, outdoor gyms) and preserving green landscapes on campus is justified not just aesthetically but for student wellness. Policies could ensure that new building projects include green design elements (like rooftop gardens or interior courtyards with natural elements) to maintain a connection to nature even in built spaces. Collaboration between student affairs, health services, and campus sustainability offices could integrate these recommendations, creating programs like “Green Mind, Healthy Mind” initiatives that combine physical activity promotion with nature engagement.

## 6 Conclusion

This study demonstrated that green exercise and nature connectedness are significant protective factors against academic burnout among university students. Green exercise was directly associated with lower emotional exhaustion, cynicism, and reduced personal accomplishment, while nature connectedness partially mediated these effects, accounting for a substantial proportion of the overall associations. Interpreted through Conservation of Resources theory and stress coping frameworks, both green exercise and nature connectedness can be viewed as interrelated resources that replenish energy, foster resilience, and mitigate strain from academic demands. These findings highlight the importance of embedding opportunities for outdoor physical activity and nature engagement into student life and campus design, suggesting that incorporating the natural dimension into higher education is a practical and sustainable strategy to promote student well-being and reduce burnout.

## Data Availability

The original contributions presented in the study are included in the article/supplementary material, further inquiries can be directed to the corresponding author.
